# WWOX CNV-67048 Functions as a Risk Factor for Epithelial Ovarian Cancer in Chinese Women by Negatively Interacting with Oral Contraceptive Use

**DOI:** 10.1155/2016/6594039

**Published:** 2016-04-11

**Authors:** Yongxiu Chen, Xiaochang Tan, Yongli Ding, Bi Mai, Xiaowen Huang, Guiying Hu, Xiping Luo

**Affiliations:** Gynecology Department, Guangdong Women and Children Hospital, No. 521, Xingnan Road, Panyu District, Guangzhou 511442, China

## Abstract

Copy number variations (CNVs) have attracted increasing evidences to represent their roles as cancer susceptibility regulators. However, little is known about the role of CNV in epithelia ovarian cancer (EOC). Recently, the CNV-67048 of WW domain-containing oxidoreductase (*WWOX*) was reported to alter cancer risks. Considering that* WWOX* also plays a role in EOC, we hypothesized that the CNV-67048 was associated with EOC risk. In a case-control study of 549 EOC patients and 571 age (±5 years) matched cancer-free controls, we found that the low copy number of CNV-67048 (1-copy and 0-copy) conferred a significantly increased risk of EOC (OR = 1.346, 95% CI = 1.037–1.747) and it determined the risk by means of copy number-dependent dosage effect (*P* = 0.009). Data from TCGA also confirmed the abovementioned association as the frequency of low copies in EOC group was 3.68 times more than that in healthy group (*P* = 0.023). The CNV also negatively interacted with oral contraceptive use on EOC risk (*P* = 0.042). Functional analyses further showed a lower mRNA level of WWOX in tissues with the 0-copy or 1-copy than that in those with the 2-copy (*P* = 0.045). Our data suggested the CNV-67048 to be a risk factor of EOC in Chinese women.

## 1. Introduction

Ovarian cancer (OC) is one of the most common tumors of genital system and causes of cancer-related death among females. In America, it was estimated that there were 21,290 new OC patients and 14,180 deaths caused by it in 2015 [1]. In China, OC ranked the tenth cancer incidence with a rate of 6.89/10^5^ in 2011 [2]. Despite the fact that treatments for OC have greatly improved these years, high mortality caused by it was still observed as above data presented. The poor prognosis depends on a late diagnosis as more than 50% of patients were diagnosed with advanced OC. Prevention is the first-rank policy to decrease OC detriment such as etiological intervention, early detection, and early diagnosis, of which the foremost step is to discriminate high risk group of OC with respect to these risk factors of OC that included both environmental and genetic factors.

To date, multiple association studies have established several genetic factors, most of which are single nucleotide polymorphisms (SNPs), to be susceptible loci for OC [3–7]. For example, three genome-wide association studies (GWASs) had revealed seven SNPs at loci 9p22, 8q24, 2q31, 19p13, 3q25, and 17q21 to be risk alleles of OC. However, as the relatively small increments in risk are exerted, these SNPs can only represent a small proportion of OC heritability, which would cause low accuracy on predicting individuals' onset risk of OC in a population [8]. Thus, to reveal missing heritability of OC is impending. Missing heritability can be explained by other variants in genomics of human, such as copy number variants (CNVs), del/ins, and DNA transversion. Among them, CNV is the second major category of genetic variants after SNP, which makes up 10%~13% of human variants [9]. More and more evidences had indicated that CNVs are associated with various cancer risks [10–14]. However, little is known about CNV on OC risk.

Recently, two studies had reported that one germline CNV-67048 that is located in a tumor suppressor gene* WW domain-containing oxidoreductase* (*WWOX*) contributed increased risks of lung cancer and gliomas in the Chinese [15, 16]. Also, CNVs across* WWOX* were reported to have potential contributions to breast cancer initiation and progression [17]. Functioning as a proapoptotic molecular and RNA-binding protein, WWOX protein plays a suppressor role in OC development [18–20]. As reported, WWOX can inhibit OC stem cells proliferation by downregulating expression of cell cycle proteins cyclin E-CDK2 and cyclin D1-CDK4 and promote cell apoptosis by upregulating expression of Wnt-5*α*, JNK, and caspase-3 [20]. WWOX also involves epithelial-mesenchymal transition of human OC stem cells [18]. Moreover, loss of WWOX expression was observed in OC, especially epithelial ovarian cancer (EOC) [21, 22]. Because previous studies showed that CNV-67048 influences WWOX expression in tumor tissues, considering the function and abnormal expression of WWOX in EOC, we hypothesized that the CNV-67048 contributes to EOC development. To test this hypothesis, we performed a case-control study including 549 EOC patients and 571 age (±5 years) matched cancer-free controls among Chinese women. We also performed functional assays to reveal the function of the CNV in EOC.

## 2. Materials and Methods

### 2.1. Study Subject

We conducted a case-control study in Guangzhou city in China. During September 2011 and July 2015, 549 EOC patients and 571 age (±5 years) matched cancer-free controls were recruited from the Guangdong Provincial Maternity and Child Care Center. EOC diagnosis is performed according to the International Federation of Gynecology and Obstetrics (FIGO). Individuals with tumor history were excluded. After a written informed consent was obtained, each participant was asked to denote 3 mL peripheral blood sample and complete a questionnaire to collect their data on sociodemographic, smoking status, alcohol consumption, menstrual and reproductive histories, and contraceptive use. This study was approved by the institutional review boards of Guangdong Provincial Maternity and Child Care Center.

### 2.2. Genotyping

Genomic DNA was extracted from 3 mL peripheral blood sample. Each DNA was then diluted into a concentration of 50 ng/*μ*L. The copy number of CNV-67048 was tested with the TaqMan® Copy Number Assay according to the protocol of Applied Biosystems by Life Technologies. Briefly, a 10 *μ*L reaction system including 1 *μ*L DNA, 5 *μ*L TaqMan Master Mix, 1 *μ*L special probes and primers for CNV-67048 (cat# Hs03922779, Applied Biosystems), 0.5 *μ*L control RNase P probe (Applied Biosystems), and 2.5 *μ*L deionized water for each subject was prepared and run on the ABI 7900 system. Then the copy number was directly calculated by the CopyCaller® Software 2.1. 5% of the samples were randomly selected to repeat genotyping and the results were 98% in agreement.

### 2.3. Bioinformatics Analysis

To validate the association between the CNV and EOC risk as well as the possible effect of the CNV on WWOX expression, we downloaded available EOC germline CNVs data and WWOX expression value of 20 Asian EOC individuals from TCGA (https://tcga-data.nci.nih.gov/tcga/). The data of Asian common CNVs in healthy controls were also downloaded from one previously published study [23].

### 2.4. WWOX mRNA Level Estimation

A total of 31 EOC and 22 normal ovarian tissues were collected from the Guangdong Provincial Maternity and Child Care Center. Total RNA was extracted using the RNAiso reagent (Takara, Japan) and then reverse-transcribed into cDNA with the PrimeScript RT Master Mix (Takara, Japan). The SYBR-Green real-time PCR was used to assess the mRNA level of WWOX in the abovementioned tissues with the primers as suggested by previously published study, 5′-TGG GTT TAC TAC GCC AAT C-3′ (forward) and 5′-GTC CGT TCT CAT CAG TTT CT-3′ (reverse) [16]. The *β*-actin was used as an internal reference. Method of 2^ΔCt^ was used to demonstrate the mRNA level of WWOX. All analyses were performed in a blinded fashion with the laboratory persons unaware of genotyping data and each assay was done in triplicate.

### 2.5. Statistical Analysis

The chi-square test was used to assess differences in the distributions of CNV-67048 copy number between EOC cases and controls. The unconditional logistic regression model with or without adjustment for surrounding factors including age, age at menarche, number of births, menstrual history, oral contraceptive use, family history of cancer, smoking status, and alcohol intake was used to infer odds ratio (OR) and 95% confidence interval (95% CI) for each association between the CNV-67048 and EOC risk. The multiplicative interaction model was used to assess the possible interaction between the CNV-67048 and selected variables on cancer risk. The Kruskal-Wallis test was used to evaluate the effect of CNV-67048 on WWOX expression in tissues. All tests were two-sided by using the IBM SPSS software (version 22.0). *P* < 0.05 was considered to be statistically significant.

## 3. Results

### 3.1. Characteristics of the Study Subjects

As shown in Table 1, age matched well between EOC cases and controls with no significant difference (*P* = 0.382). Also, there was no significant difference in frequency distribution of menstrual history, family history of cancer, smoking status, and alcohol intake between the two groups (*P* > 0.05 for all). However, significantly higher frequency of menarche age less than 15 years, births number no less than 4, and reported null oral contraceptive use were observed in EOC cases than in controls (*P* values are 0.039, <0.001, and 0.039 in turn). Moreover, EOC cases were more likely to be heavy smokers with no less than 20 pack-years smoked than controls (*P* = 0.029).

### 3.2. Contribution of* WWOX* CNV-67048 to EOC Development

As shown in Table 2, three types of copy number of CNV-67048, which are 2-copy, 1-copy, and 0-copy, were detected. The alter frequency of loss allele in the current study (10.3%) was equivalent to that in Asian individuals as reported (10.0%) [23]. The CNV was related to EOC susceptibility as its frequency distributions of copy number were significantly different between EOC cases and controls (*P* = 0.005). Results from the unconditional logistic regression model without adjustment for surrounding factors presented significant increases in EOC risk in both 1-copy carriers (OR = 1.325, 95% CI = 1.024–1.714) and 0-copy carriers (OR = 2.425, 95% CI = 1.261–4.665) compared to 2-copy carriers. A tendency for an increased EOC risk was further observed accompanied by decreased copy number (*P* = 0.002). Moreover, after adjustment for surrounding factors, the 0-copy (OR = 2.198, 95% CI = 1.111–4.348) and a combination of 1-copy and 0-copy (OR = 1.346, 95% CI = 1.037–1.747) still conferred significantly increased risk of EOC. The tendency is also significant (*P* = 0.009). However, we did not find any statistically significant associations between CNV-67048 and EOC stages in case only study (Table 2). In addition, in order to validate our result, we compared the Asian germline CNVs data from TCGA database of EOC and Asian common CNVs data of health population as published [23]; the frequency of low copy number in EOC group was 3.68 times more than that in healthy group with statistical significance (*P* = 0.023).

### 3.3. Associations between CNV-67048 and EOC Risk Stratified by Selected Variables

**Figure 1:**
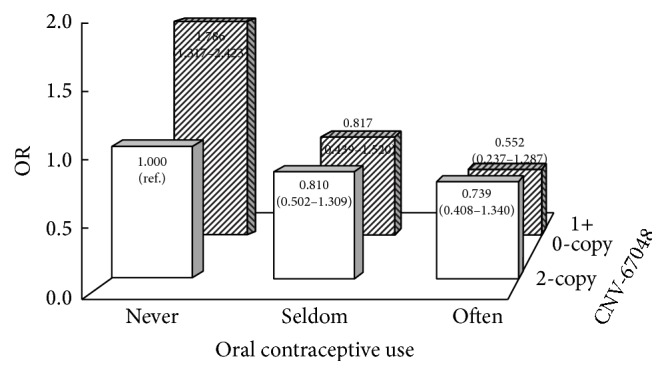
Negative interaction between the CNV-67048 and oral contraceptive use on EOC risk. The subjects who carried 2-copy CNV-67048 and never used oral contraceptive were defined as reference. The loss copy of CNV-67048 significantly interacted with null oral contraceptive use on EOC risk.

As shown in Table 3, the contributions of CNV-67048 on EOC risk were only significant in subgroups of 1 to 3 births' number, null oral contraceptive use, no family history of cancer, never smokers, heavy smokers, and never drinkers. However, the nonsignificant effect in the corresponding subgroups may be due to the limited sample size. Interestingly, the interaction analysis showed that the CNV-67048 significantly interacted with oral contraceptive use on EOC risk. As the CNV exerted a risk effect and oral contraceptive use exhibited a protective effect on EOC, their interactions are negative (*P* = 0.042). We further used the additive interaction model to show detailed interaction effect between them in Figure 1. As shown, compared to subjects who carried 2-copy CNV-67048 and never used oral contraceptive, those who carried 1-copy or 0-copy and never used oral contraceptive harbored the highest EOC risk (OR = 1.786, 95% CI = 1.317–2.423).

### 3.4. Effect of the CNV-67048 on WWOX Expression

**Figure 2:**
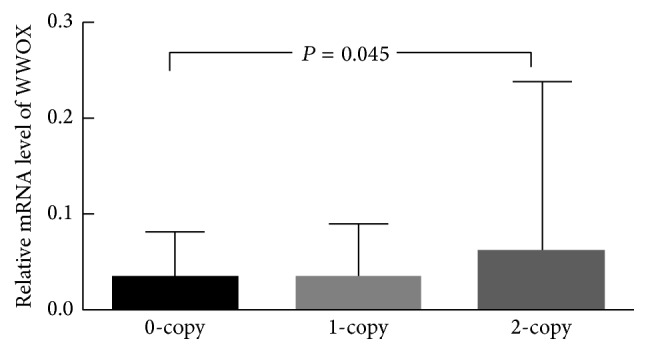
Effect of the CNV-67048 on WWOX mRNA expression in ovary tissues. The Kruskal-Wallis test was used to evaluate the effect of CNV-67048 on WWOX expression in tissues.

As shown in Figure 2, in the total 53 cases of ovarian tissues, the mRNA levels of WWOX were significantly lower in tissues with the 0-copy (median: 0.0342) or 1-copy (median: 0.0347) of CNV-67048 than in those with the 2-copy (median: 0.0617; *P* = 0.045). We also queried the TCGA database and downloaded the 20 cases of Asians gene expression data; the gene expression of* WWOX* was also higher in tissues with 2-copy than in those with 0-copy or 1-copy. The difference between them is 0.082 (2-copy, 0.725 ± 0.100, versus 0-copy and 1-copy, 0.643 ± 0.053).

## 4. Discussion

In the current study, we found that the CNV-67048 of* WWOX* was significantly related to the risk of EOC in Chinese women. The CNV also negatively interacted with oral contraceptive use, because it could significantly tarnish the protective role of oral contraceptive use on EOC development. However, this study did not show any significant association between the CNV-67048 and EOC stages.

The WWOX protein is a kind of broad-spectrum tumor suppressor involving many kinds of human cancers [24]. Functional suppression of WWOX prevents apoptotic cell death induced by a variety of stress stimuli, such as tumor necrosis factor, UV radiation, and chemotherapeutic drug treatment [25]. Through protein-protein interaction, WWOX could directly bond onto a lot of well-known cancer-related molecules such as the p53, p73, Jun, and ErbB4 to enhance apoptosis [26]. WWOX also participates in the cellular metabolism and affects tumor metabolism and thus inhibits tumorigenesis [27]. Alteration of WWOX has been observed in many tumors, including breast [28], ovarian [22], prostate [29], lung [16], hepatocellular [30], gastric [31], and other cancers [32–35], and loss or reduction of its expression is reported to be correlated with worse clinical prognosis such as breast and ovarian cancer [28, 36]. Similarly in EOC, lower WWOX expression was found in tumors compared to normal ovaries [22]. Previous studies had showed that the 0-copy or 1-copy could cause a lower WWOX expression in human tissues than the 2-copy; thus individuals carrying low copy might be more predisposed to develop EOC in response to carcinogenic stimulation than individuals carrying 2-copy because of their innate differences in WWOX expression.

We also found a negative interaction between the CNV-67048 and oral contraceptive use on EOC risk. It is well-established that oral contraceptive plays a protective role in EOC risk [37]. Oral contraceptive can inhibit ovulation frequency and thus decrease the risk of EOC, because chronic stimulus caused by several ovulations can result in abnormal cell proliferation and repair and further tumorigenesis. As mentioned above, WWOX participates in the cell process in response to such stimulus and plays a role in cell repair. Thus, it is possible that the low expression of WWOX driven by low copy number of CNV-67048 enhances the cellular malignant change and suppresses the protective effect of oral contraceptive use.

*WWOX* CNV-67048 has been reported to be associated with lung cancer and gliomas risk in previous studies [15, 16]. Here, consistently it was found to be related to EOC risk. However, although loss of WWOX expression correlates with advanced EOC stages as reported, we did not find that the CNV was correlated with the stages. By now, only four studies have tested the effect of CNV on EOC development in large populations. Gonzalez Bosquet et al. used the integrating high-throughput data from TCGA including CNVs to construct a molecular signature to predict chemoresponse in EOC [38]. Kamieniak et al. presented a CNV hallmark of BRCA1 and BRCA2 EOC [39]. Huang et al. identified special copy number landscapes for EOC histotypes [40]. Fridley et al. tested genome-wide CNVs but found no association between inherited CNVs and ovarian cancer survival [41]. It is to be observed that these above studies did not study the effect of CNV on EOC risk. To the best of our knowledge, our study is the first study to show that a CNV is related to the risk of EOC.

Although this case-control study presented a significant association between the* WWOX* CNV-67048 and EOC risk and got TCGA support to be reliable and functional, there were some unavoidable limitations in the current study. As a hospital-based case-control study, there must be a selection bias such as Berkson bias and an information bias including recall bias. Meanwhile, our study sample size is relatively small. Additionally, for those interviews completed, data on some variables were not obtained or were unclear. This may cause bias on the estimation of the abovementioned association. Thus, further study with large sample size in other ethnics is warranted.

In conclusion, our data revealed the CNV-67048 and its interaction with oral contraceptive use to be in association with EOC risk in Chinese women. This CNV might be a genetic risk factor of EOC in Chinese women.

## Figures and Tables

**Table 1:** Frequency distributions of selected variables among EOC cases and controls.

Variables	Case (*n* = 549) *n* (%)	Control (*n* = 571) *n* (%)	*P* ^a^
Age (years)			
≤55	277 (50.46)	303 (53.06)	0.382
>55	272 (49.54)	268 (46.94)	
Age at menarche (years)			
<15	211 (53.55)	257 (46.73)	0.039^*∗*^
≥15	183 (46.45)	293 (53.27)	
Unclear	155 (—)	21 (—)	
Number of births			
0	16 (2.91)	11 (1.93)	<0.001
1–3	409 (74.50)	490 (85.81)	
≥4	124 (22.59)	70 (12.26)	
Menstrual history			
Premenopause	152 (27.69)	131 (22.94)	0.068
Menopause	397 (72.31)	440 (77.06)	
Oral contraceptive use			
Never	414 (82.31)	399 (75.86)	0.039^*∗*^
Seldom	56 (11.13)	78 (14.83)	
Often	33 (6.56)	49 (9.32)	
Unclear	46 (—)	45 (—)	
Family history of cancer			
Yes	40 (7.29)	38 (6.65)	0.678
No	509 (92.71)	533 (93.35)	
Smoking status			
Ever	68 (12.39)	54 (9.46)	0.116
Never	481 (87.61)	517 (90.54)	
Pack-years smoked			
≥20	36 (6.56)	18 (3.15)	0.029
<20	32 (5.83)	36 (6.30)	
0	481 (87.61)	517 (90.54)	
Alcohol intake			
Ever	34 (6.19)	49 (8.58)	0.127
Never	515 (93.81)	522 (91.42)	
Staging			
I + II	43 (7.84)		
III	420 (76.50)		
IV	86 (15.66)		

^a^ *P* values for a χ^2^ test.

^*∗*^Statistical analysis excluded subjects with unclear or unknown data.

**Table 2:** Associations between *WWOX* CNV-67048 copy numbers and EOC risk and stages.

CNV-67048 genotypes	*n* (%)	*n* (%)	*P*	Crude OR (95% CI)	Adjusted OR (95% CI)^a^
*Case-control study*	*Patients*	*Controls*			
Total number	549	571			
2-copy	339 (61.75)	397 (69.53)	0.005	1.000 (ref.)	1.000 (ref.)
1-copy	181 (32.97)	160 (28.02)		1.325 (1.024–1.714)	1.268 (0.967–1.663)
0-copy	29 (5.28)	14 (2.45)		2.425 (1.261–4.665)	2.198 (1.111–4.348)
1 + 0-copy	210 (38.25)	174 (30.47)		1.413 (1.103–1.811)	1.346 (1.037–1.747)
Trend test *P* value				0.002	0.009

*Case only study*	*Stages III + IV*	*Stages I + II*			
Total number	506	43	0.308		
2-copy	309 (61.07)	30 (69.77)		1.000 (ref.)	1.000 (ref.)
1-copy	168 (33.20)	13 (30.23)		1.255 (0.637–2.470)	1.330 (0.655–2.701)
0-copy	29 (5.73)	0 (0.00)		—	—
1 + 0-copy	197 (38.93)	13 (30.23)		1.471 (0.749–2.888)	1.554 (0.771–3.129)
Trend test *P* value				0.129	0.112

^a^Adjusted in a logistic regression model that included age, age at menarche, number of births, menstrual history, oral contraceptive use, family history of cancer, smoking status, and alcohol intake.

**Table 3:** Associations between CNV-67048 and EOC risk stratified by selected variables.

	EOC Patients (*n* = 549)		Controls (*n* = 571)		Adjusted OR (95% CI)^a^	*P*_inter_^b^
	2-copy *n* (%)	1 + 0-copy *n* (%)	2-copy *n* (%)	1 + 0-copy *n* (%)	1 + 0-copy versus 2-copy	
Age (years)						
≤55	177 (63.90)	100 (36.10)	206 (67.99)	97 (32.01)	1.175 (0.817–1.692)	0.396
>55	162 (59.56)	110 (40.44)	191 (71.27)	77 (28.73)	1.474 (0.990–2.194)	
Age at menarche (years)						
<15	133 (63.03)	78 (36.97)	177 (68.87)	80 (31.13)	1.315 (0.878–1.971)	0.564
≥15	124 (67.76)	59 (32.24)	207 (70.65)	86 (29.35)	1.193 (0.775–1.839)	
Number of births						
0	10 (62.50)	6 (43.75)	7 (63.64)	4 (36.36)	0.414 (0.043–4.002)	0.075
1–3	238 (58.19)	171 (41.81)	342 (69.80)	148 (30.20)	1.556 (1.164–2.081)	
≥4	91 (73.39)	33 (26.61)	48 (68.57)	22 (31.43)	0.873 (0.442–1.722)	
Menstrual history						
Premenopause	89 (58.55)	63 (41.45)	92 (70.23)	39 (29.77)	1.451 (0.788–2.671)	0.563
Menopause	250 (62.97)	147 (37.03)	305 (69.32)	135 (30.68)	1.262 (0.931–1.710)	
Oral contraceptive use						
Never	239 (57.73)	175 (42.27)	283 (70.93)	116 (29.07)	1.825 (1.348–2.471)	0.042
Seldom	35 (62.50)	21 (37.50)	51 (65.38)	27 (34.62)	1.019 (0.454–2.291)	
Often	23 (69.70)	10 (30.30)	33 (67.35)	16 (32.65)	0.464 (0.110–1.953)	
Family history of cancer						
Yes	23 (57.50)	17 (42.50)	25 (65.79)	13 (34.21)	2.440 (0.826–7.208)	0.654
No	316 (62.08)	193 (37.92)	372 (69.79)	161 (30.21)	1.333 (1.016–1.748)	
Smoking status						
Ever	43 (63.24)	25 (36.76)	37 (68.52)	17 (31.48)	1.353 (0.513–3.573)	0.488
Never	296 (61.54)	185 (38.46)	360 (69.63)	157 (30.37)	1.528 (1.165–2.003)	
Pack-years smoked						
≥20	23 (63.89)	13 (36.11)	14 (77.78)	4 (22.22)	10.067 (1.110–91.293)	0.695
<20	20 (62.50)	12 (37.50)	23 (63.89)	13 (36.11)	0.676 (0.175–2.616)	
0	296 (61.54)	185 (38.46)	360 (69.63)	157 (30.37)	1.528 (1.165–2.003)	
Alcohol intake						
Ever	24 (70.59)	10 (29.41)	36 (73.47)	13 (26.53)	1.548 (0.383–6.248)	0.455
Never	315 (61.17)	200 (38.83)	361 (69.16)	161 (30.84)	1.401 (1.070–1.835)	

^a^Adjusted in a logistic regression model that included age, age at menarche, number of births, menstrual history, oral contraceptive use, family history of cancer, smoking status, and alcohol intake.

^b^ *P* value from a multiple interaction analysis.
